# A Polycistronic tRNA-amiRNA System Reveals the Antiviral Roles of *NbAGO1a/1b/2* Against *Soybean mosaic virus* Infection

**DOI:** 10.3390/plants14243724

**Published:** 2025-12-06

**Authors:** Wenhua Bao, Danyang Sun, Yan Qiu, Xiaoke Zhao, Hada Wuriyanghan

**Affiliations:** 1 College of Life Science and Technology, Inner Mongolia Normal University, Hohhot 010022, China; 20244015046@mails.imnu.edu.cn (D.S.); 20254015014@mails.imnu.edu.cn (Y.Q.); 20254015057@mails.imnu.edu.cn (X.Z.); 2 Key Laboratory of Biodiversity Conservation and Sustainable Utilization in Mongolian Plateau for College and University of Inner Mongolia Autonomous Region, Hohhot 010022, China; 3 Key Laboratory of Forage and Endemic Crop Biotechnology, Ministry of Education, School of Life Sciences, Inner Mongolia University, Hohhot 010070, China; nmhadawu77@163.com

**Keywords:** RNA interference, Argonaute protein, *Soybean mosaic virus*, *Nicotiana benthamiana*, Artificial miRNA, Polycistronic tRNA-amiRNA (PTA)

## Abstract

RNA interference (RNAi) is a crucial antiviral defense mechanism in plants, where Argonaute (AGO) proteins play a central role. However, the function of AGO proteins in the interaction between *Soybean mosaic virus* (SMV) and *Nicotiana benthamiana* remains unclear. In this study, SMV pathogenicity was confirmed using an SMV-GFP infectious clone, with typical symptoms and systemic GFP fluorescence observed 14 days post-inoculation. Real-time quantitative reverse transcription polymerase chain reaction analysis revealed dynamic regulation of multiple *NbAGO* genes upon infection. Notably, *NbAGO1a*, *NbAGO1b*, and *NbAGO2* were significantly upregulated and positively correlated with viral accumulation, suggesting their critical roles in antiviral defense. Based on these findings, these three genes were selected as targets for artificial microRNA (amiRNA) silencing. Three amiRNAs were designed for each gene using the *Arabidopsis* miR1596 backbone, with the most effective sequences exhibiting silencing efficiencies ranging from 75.2% to 98.1%. A polycistronic tRNA-amiRNA (PTA) cassette was constructed using Golden Gate cloning technology to simultaneously target all three genes. Co-infection assays indicated that the PTA cassette enhanced SMV accumulation more effectively than single amiRNAs, as evidenced by increased GFP fluorescence (49.1–60.5%) and pronounced leaf necrosis. The PTA system downregulated the expression of *NbAGO1a*, *NbAGO1b*, and *NbAGO2* by 18.4–26.7%. Furthermore, silencing *NbAGO2* alone resulted in severe necrosis, underscoring its essential role in this antiviral defense mechanism. This study elucidates the importance of *NbAGO1a*, *NbAGO1b*, and *NbAGO2* in antiviral immunity and demonstrates the utility of the PTA system for efficient multi-gene silencing, offering valuable insights for developing RNAi-based antiviral strategies.

## 1. Introduction

Over millions of years of coevolution with pathogens, plants have developed a complex and sophisticated defense system. Among the various defense mechanisms, RNA interference (RNAi) plays a crucial role in plant immunity as a highly specific and adaptable antiviral strategy [1]. RNAi inhibits viral replication and spread by mediating sequence-specific gene silencing, resulting in the precise degradation of viral RNA. This process primarily involves four key steps: (1) Exogenous or endogenous double-stranded RNA (dsRNA) activates the silencing signal. (2) RNase III-type enzymes, Dicer or Dicer-like (DCL), cleave the dsRNA into small interfering RNAs (siRNAs) that are 18–25 nucleotides (nt) in length. (3) The antisense strand of the siRNA assembles with proteins such as Argonaute (AGO) to form the RNA-induced silencing complex (RISC). (4) Guided by the siRNA, RISC recognizes complementary mRNA sequences and executes target silencing through AGO-mediated cleavage or translational repression [2]. In plants, the antiviral RNAi mechanism primarily operates via two pathways: Post-transcriptional gene silencing (PTGS) and RNA-directed DNA methylation (RdDM). In the PTGS pathway, virus-derived dsRNA is processed by DCL enzymes into 21–22 nt viral siRNAs (vsiRNAs), which are then loaded into AGO1 or AGO2 to form an active RISC that directly cleaves viral RNA. In the RdDM pathway, 24 nt vsiRNAs generated by DCL mediate DNA methylation through AGO4, thereby suppressing viral gene expression at the transcriptional level [3]. Early genetic analyses revealed that *Arabidopsis* ago1 mutants exhibited high susceptibility to *Cucumber Mosaic Virus* (CMV), which first uncovered the antiviral function of plant AGO proteins [4]. Subsequent studies have demonstrated that multiple members of the *Arabidopsis* AGO family possess antiviral activity, including AGO1, AGO2, AGO3, AGO4, AGO5, AGO7, and AGO10. These AGO proteins exhibit specificity against certain viruses [5]. For example, in *Arabidopsis*, AGO1 is essential for resistance against *Turnip Crinkle Virus* (TCV) and *Cucumber Mosaic Virus* (CMV) and is considered a key player in the siRNA pathway [6]. Further studies confirmed that AGO1 is a primary executor of plant defenses against various viruses. Notably, in some plants such as *Oryza sativa* and *N. benthamiana*, the *AGO1* gene has duplicated during evolution, resulting in multiple paralogous genes *AGO1a-1d* in *Oryza sativa*; *AGO1a* and *AGO1b* in *N. benthamiana* [6]. Research suggests that these homologs exhibit functional redundancy and a degree of specificity, collectively forming the first line of defense in the plant’s basic antiviral network. However, to effectively colonize their hosts, viruses often produce viral suppressors of RNA silencing that target AGO1 proteins, allowing them to evade host immunity [7]. In response, plants have developed compensatory mechanisms, with AGO2 being a key representative. Studies indicate that AGO2 is activated to help maintain antiviral capacity when AGO1 function is compromised. Additionally, AGO2 is involved in systemic signaling and in novel defense pathways such as cross-kingdom RNAi [1]. Consequently, the synergistic and backup network formed between AGO1 and AGO2 significantly enhances the robustness of plant immunity. Based on this background, this study focuses on key AGO members in *N. benthamiana* NbAGO1a, NbAGO1b, and NbAGO2 to explore their specific roles and collaborative mechanisms in antiviral defense.

The artificial microRNA (amiRNA) technology has emerged as a vital tool for functional gene research, allowing for precise regulation of target gene expression. This technology involves modifying the backbone of endogenous miRNA precursors by replacing the original miRNA/miRNA* region with artificially designed amiRNA/amiRNA* sequences. This process generates small RNAs with novel targeting functions in vivo [8]. The amiRNA technology offers several advantages, including high specificity, low off-target effects, and design flexibility. It has been widely utilized in crop breeding, stress resistance research, and gene functional analysis [9]. To enhance silencing efficiency and enable coordinated regulation of multiple genes, this study introduces a polycistronic tRNA-amiRNA (PTA) expression system. This system employs Golden Gate cloning technology to assemble multiple tRNA-amiRNA units into a single expression cassette. After transcription, RNase P and RNase Z cleave the cassette in vivo, releasing multiple amiRNAs that enable efficient and simultaneous silencing of different target genes [10].

This study systematically analyzed the development of symptoms following *Soybean mosaic virus* (SMV) infection in *N. benthamiana*, as well as the expression dynamics of *NbAGO* genes. In addition, it examined the roles of both single-gene silencing using amiRNA and a multi-target PTA silencing system in modulating the RNAi pathway and SMV resistance. Experimental results confirmed successful SMV infection and induction of characteristic symptoms in *N. benthamiana*. The study revealed differential expression patterns of *NbAGO1a*, *NbAGO1b*, and *NbAGO2* under SMV stress, clarifying their crucial roles in the antiviral pathway. Additionally, the study successfully constructed an efficient multi-target PTA silencing system, providing important theoretical and technical support for a deeper understanding of the antiviral mechanisms mediated by the plant RNAi pathway. This will aid in developing novel strategies for crop disease control.

## 2. Results

### 2.1. Symptoms of SMV-GFP Infection and Expression Response of NbAGOs Genes in Nicotiana benthamiana

**Figure 1:**
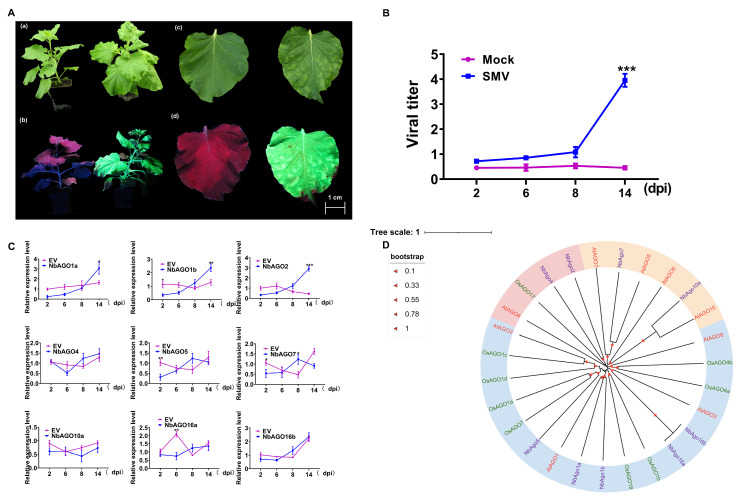
Phenotype *of N*. *benthamiana* infected with SMV and expression levels of *NbAGO* genes in systemic leaves at different infection stages. (**A**) Phenotype of *N. benthamiana* infected with SMV-GFP at 14 dpi. (**a**) Whole plant phenotype under natural light. (**b**): Whole plant phenotype under UV light, with GFP visualized using a handheld UV lamp (wavelength = 365 nm). (**c**,**d**) Magnified views of viral symptoms on the leaves. (**B**) Quantification of SMV RNA accumulation by real-time quantitative reverse transcription polymerase chain reaction (RT-qPCR) in systemic leaves of SMV-GFP inoculated *N. benthamiana* at various time points post-infection. (**C**) Relative expression levels of nine *NbAGO* genes (*NbAGO1*, *NbAGO1b*, *NbAGO2*, *NbAGO4*, *NbAGO5*, *NbAGO6*, *NbAGO7*, *NbAGO10a*, *NbAGO16a*, and *NbAGO16b*) in the systemic leaves of SMV-infected *N. benthamiana* at different infection stages, as determined by RT-qPCR. Plants inoculated with empty pBI121 vector (EV) served as control treatment. Target genes expression in control was arbitrarily designated as 1. The bars represent SD of three independent biological replicates, each with three repeats, *: *p* < 0.05, **: *p* < 0.01, ***: *p* < 0.001. (**D**) Phylogenetic analysis of AGO proteins from *Arabidopsis thaliana* (At), *Oryza sativa* (Os), and *N. benthamiana* (Nb). In the phylogenetic tree, blue, pink, and red represent three different subfamilies of the AGO family, respectively.

To evaluate the infectivity of the SMV-GFP infectious clone that was previously constructed, infection assays were conducted on *N. benthamiana*. At 14 days post-inoculation (dpi), the plants inoculated with SMV-GFP displayed noticeable phenotypic changes, including mosaic patterns, curling, and distortion. Under UV light, GFP fluorescence signals were observed in the systemic leaves, confirming the successful spread of the virus. To quantify the dynamics of viral accumulation, apical systemic leaves from three plants were collected at 2, 6, 8, and 14 dpi for viral titer detection. There was a significant increase in SMV accumulation in the systemic leaves beginning at 8 dpi, with viral load progressively rising over time (Figure 1A). These findings confirm that SMV effectively infects *N. benthamiana* and induces typical symptoms.

To investigate the response of *NbAGOs* genes to SMV infection and to characterize their expression dynamics during various stages of infection, we collected apical systemic leaves from three plants at 2, 6, 8, and 14 dpi for RT-qPCR analysis of NbAGOs expression levels (Figure 1B). At 2 dpi, expression levels of *NbAGO1a*, *NbAGO1b*, *NbAGO2*, *NbAGO*5, *NbAGO7*, and *NbAGO10* in SMV-infected plants were significantly downregulated compared to plants treated with a mock solution. As infection progressed (6–14 dpi), expression levels of these genes gradually returned to near-mock levels. Notably, *NbAGO1a*, *NbAGO1b*, and *NbAGO2* exhibited a consistent expression pattern in SMV-infected plants, with all three genes demonstrating rapid upregulation from low expression at 2 dpi to significantly higher levels than mock by 14 dpi (Figure 1C). Notably, the expression trends of *NbAGO1a*, *NbAGO1b*, and *NbAGO2* closely mirrored the temporal pattern of SMV accumulation in the systemic leaves, as illustrated in Figure 1B.

In summary, the genes involved in the *N. benthamiana* RNAi pathway, including *NbAGO1a*, *NbAGO1b*, *NbAGO2*, *NbAGO5*, *NbAGO7*, *NbAGO10*, and *NbAGO16*, exhibited differential responses to SMV infection. Among these, *NbAGO1a*, *NbAGO1b*, and *NbAGO2* displayed significant upregulation, which was positively correlated with the accumulation of SMV. This suggests that these three AGO proteins play critical roles in the pathogenesis of SMV. Consequently, we selected *NbAGO1a*, *NbAGO1b*, and *NbAGO2* as target genes for further investigation.

To elucidate the phylogenetic relationships of AGO proteins in *N*. *benthamiana*, we performed multiple sequence alignment and constructed a phylogenetic tree using the neighbor-joining method in MEGA-X (Version 10.1.7) software with nine *AGO* genes from *N. benthamiana* (NbAGOs), *Arabidopsis thaliana* (AtAGOs), and *Oryza sativa* (OsAGOs). Phylogenetic analysis revealed that these AGO proteins can be classified into three evolutionarily conserved subfamilies, which closely correspond to the well-defined functional clades established in *Arabidopsis*. Specifically: (1) *N. benthamiana* NbAGO1a, NbAGO1b, NbAGO5, NbAGO16a, and NbAGO16b clustered with the *Arabidopsis* AGO1/5/10 clade, which is primarily involved in post-transcriptional gene silencing and plant development regulation; (2) NbAGO7 and NbAGO10a grouped with the *Arabidopsis* AGO2/3/7 clade, known for its roles in disease resistance (e.g., antiviral defense) and the production of trans-acting siRNAs; (3) NbAGO4 and NbAGO2 clustered with the *Arabidopsis* AGO4/6/8/9 clade and *Oryza sativa* OsAGO17, a clade mainly associated with RNA-directed DNA methylation and transcriptional-level gene silencing (Figure 1D). These findings not only demonstrate a close phylogenetic relationship among the AGO gene families of the three species but also suggest that *N. benthamiana* AGO members may have retained functional roles analogous to their *Arabidopsis* homologs.

### 2.2. Transient Silencing of Target Genes Using Specific amiRNA in N. benthamiana

**Figure 2:**
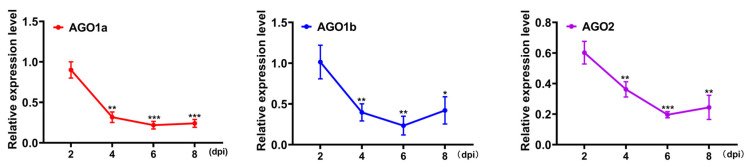
Silencing of *NbAGO1a*, *NbAGO1b* and *NbAGO2* via transient expression of amiRs from pBI121-based vector. pBI121-NbAGO1a-amiR1, pBI121-NbAGO1b-amiR1 and pBI121-NbAGO2-amiR1 construct were transformed into GV3101, infiltrated on *N. benthamiana* leaves, and the leaves were collected at different dpi. Genes expression was determined by quantitative real-time PCR. Plants inoculated with empty pBI121 vector (EV) served as control treatment. Genes expression in control was arbitrarily designated as 1. *NbActin* gene served as the internal control. Significant differences between control and treatment groups were calculated by Student’s *t*-test. Error bars represent the standard deviation (SD) of three independent biological replicates, each with three repeats, *: *p* < 0.05, **: *p* < 0.01, ***: *p* < 0.001.

In this study, we focused on three crucial genes involved in the RNAi pathway in *N. benthamiana*: *NbAGO1a*, *NbAGO1b*, and *NbAGO2*. For each gene, three different amiRNA sequences were designed, and pre-miRNA sequences were amplified utilizing *Arabidopsis* miR1596 backbones as a template. This resulted in the construction of a pBI121 expression binary vector. After confirming successful cloning through colony PCR, double enzyme digestion, and sequencing, we constructed nine recombinant vectors. These vectors were then transformed into *Agrobacterium* GV3101 to prepare an infection solution that was injected into the leaves of wild-type *N. benthamiana*. To determine the optimal time point for gene silencing, the recombinant vectors expressing amiRNA1 for each of the three *AGO* genes were used in infection experiments. Samples were collected at 2, 4, 6, and 8 dpi, respectively, and the expression levels of *AGO* genes were analyzed using RT-qPCR. The most significant gene silencing effect was observed at 6 dpi. Consequently, for the subsequent evaluation of the other amiRNA sequences, samples were consistently collected six days after agroinfiltration (Figure 2).

**Figure 3:**
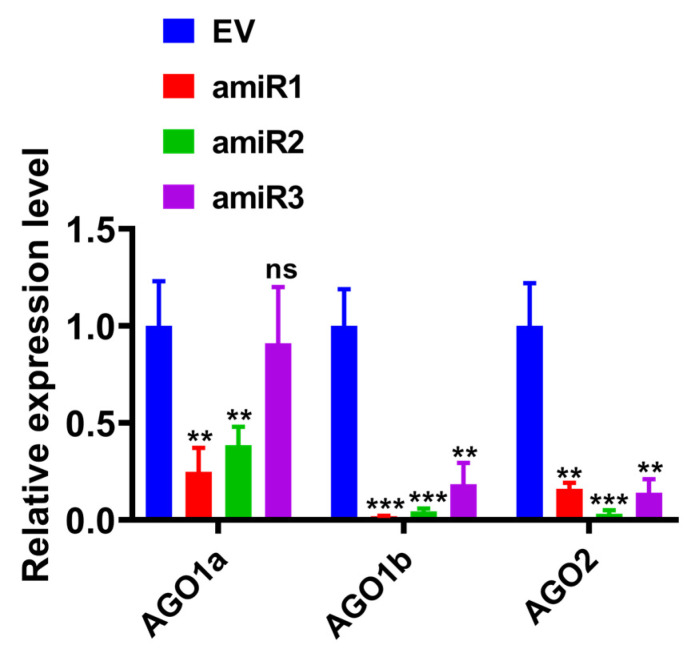
Effects of three amiRNA sequences on target gene silencing were investigated using pBI121-based expression vectors that encoded amiRNA1, amiRNA2, and amiRNA3. These vectors were utilized for the transient silencing of *NbAGO1a*, *NbAGO1b*, and *NbAGO2* genes in *N*. *benthamiana*. Each constructed pBI121-amiRNA vector was transformed into *Agrobacterium tumefaciens* GV3101 and then infiltrated into *N. benthamiana* leaves. Leaf samples were collected six days after infiltration for analysis. The expression levels of the target genes were measured using RT-qPCR. Leaves infiltrated with the empty pBI121 vector (EV) were used as the control group. The *NbActin* gene was used as an internal reference for normalization. Significant differences between treatment groups and the control were determined using Student’s *t*-test, **: *p* < 0.01, ***: *p* < 0.001.

The nine constructed amiRNA recombinant vectors were used to individually infect *N. benthamiana* plants via agroinfiltration. Leaf samples were collected at six dpi and subjected to RT-qPCR analysis to evaluate and compare the silencing efficiencies of the three amiRNAs designed for each target gene. In the interference assay, targeting the amiRNA1 of the *NbAGO1a* gene exhibited the most pronounced silencing effect, reducing the target gene expression by 75.2%. This was notably higher than the reductions achieved by amiRNA2 (61.5%) and amiRNA3 (9%). For *NbAGO1b*, all three amiRNAs demonstrated high interference efficiency, with silencing rates of 98.1% for amiRNA1, 95.6% for amiRNA2, and 81.5% for amiRNA3. In the case of *NbAGO2*, the highest silencing efficiency was observed with amiRNA2 (96.8%), followed by amiRNA3 (86%) and amiRNA1 (84%). Consequently, the most effective silencing sequences for the three genes were identified as NbAGO1a-amiRNA1, NbAGO1b-amiRNA1, and NbAGO2-amiRNA2 (Figure 3).

### 2.3. Constructing AGO Gene-Specific PTA Expression Cassettes of N. benthamiana

To further enhance RNAi efficiency, a multi-target gene silencing system was developed based on PTA expression cassettes [11]. It utilized the Golden Gate cloning method, which employs type II restriction endonuclease, such as *Bsa*I, to cleave DNA and form sticky ends. Meanwhile, T_7_ ligase facilitates the connection of various target DNAs in a specific order, allowing for gapless assembly. After PTA transcription, tRNA was identified and cleaved with RNaseP and RNaseZ specificity, leading to multiple amiRNA-interfering relevant target genes simultaneously. We anticipated a more significant gene silencing effect post-transcription when compared to using single amiRNAs (Figure 3).

**Figure 4:**
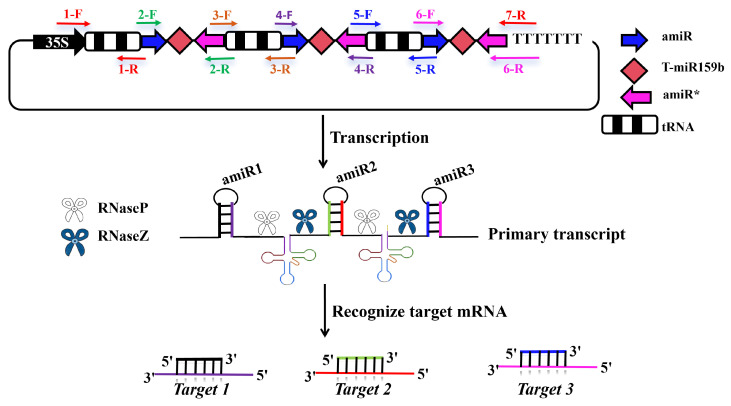
Construction and mechanism of the PTA expression cassette. The arrows indicate the forward and reverse primers for amplification of each fragment. Specifically, the 1-F and 1-R represent the forward and reverse primers for amplifying the first tRNA, while the 2-F and 2-R represent the forward and reverse primers for amplifying the first amiRNA (NbAGO1a-amiRNA1). Additionally, the 3-F and 3-R represent the forward and reverse primers for amplifying the second tRNA. The 4-F and 4-R represent the forward and reverse primers for amplifying the second amiRNA (NbAGO1b-amiRNA1), whereas 5-F and 5-R represent the forward and reverse primers for amplification of the third tRNA. Furthermore, 6-F and 6-R represent the forward and reverse primers for amplification of the third amiRNA (NbAGO2-amiRNA2), and 1-F and 7-R represent the forward and reverse primers for amplification of the entire PTA expression cassette.

We selected *NbAGO1a*, *NbAGO1b*, and *NbAGO2* in *N. benthamiana* as target genes. Based on the optimal RNAi efficiency verified for each target, we designed amiRNA sequences and incorporated them into the PTA expression cassettes. Utilizing *Arabidopsis* miR159b backbones and pGTR plasmids as templates, three pre-miRNAs and tRNA sequences were amplified, respectively. Notably, tRNA1 contains a guide RNA scaffold sequence in proximity to the primer to prevent the primers from annealing to other medium tRNA sequences during long-fragment amplification. Additionally, the first forward primer functions as the forward primer that ultimately amplifies the full-length PTA expression cassette (Figure 4).

**Figure 5:**
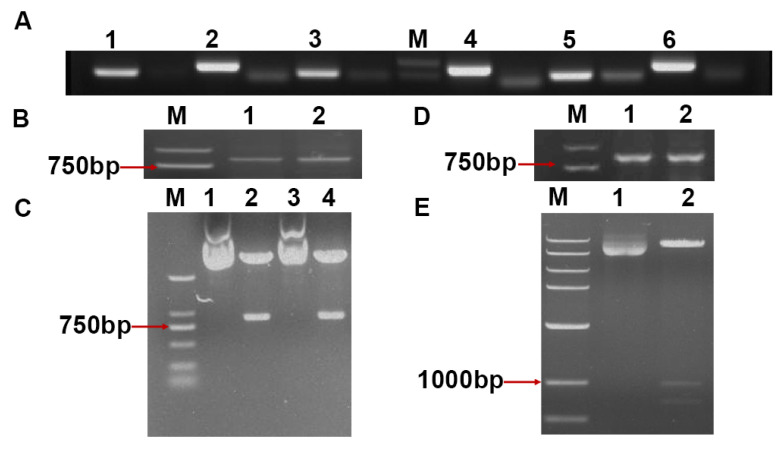
Construction of recombinant PTA expression cassette vectors. (**A**) In Lanes 1, 3, and 5, three tRNA fragments were amplified using pGTR as the template, with sizes of tRNA1 (124 bp), tRNA2 (120 bp), and tRNA3 (120 bp), respectively. In Lanes 2, 4, and 6, three amiRNA sequences were amplified using T-miR159b as the template, with sizes of NbAGO1a-amiRNA1 (187 bp), NbAGO1b-amiRNA1 (187 bp), and NbAGO2-amiRNA2 (210 bp), respectively. (**B**,**C**) PCR analysis of the PTA expression cassette after Golden Gate assembly, and restriction enzyme digestion verification of the recombinant cloning vector pMD19-T-PTA-AGO. (**D**) PCR analysis of the pBI121-AGO recombinant plasmid. (**E**) Double restriction enzyme digestion analysis of the pBI121-PTA-AGO recombinant plasmid. Lane 1: Undigested recombinant plasmid pBI121-PTA-AGO; Lane 2: Digestion products of pBI121-PTA-AGO after double digestion with *Xba*Ι and *kpn*Ι.

PCR amplification fragments for the three amiRNAs and three tRNAs used to construct the PTA-AGO expression cassette corresponded with the expected sizes (Figure 5A). Using the Golden Gate cloning method, we assembled the three amiRNAs and three tRNAs in a predetermined order confirmed by PCR, restriction enzyme digestion, and sequencing. The PTA expression framework of the corresponding target gene was obtained via PCR amplification, gel-extracted, and subsequently ligated into the pBI121 vector to construct the recombinant plasmid pBI121-PTA-AGO. PCR and restriction digestion results indicated specific bands within the 750–1000 bp range (845 bp), while sequencing results verified that the sequence matched the expected results. These findings confirm that the PTA-AGO expression framework was successfully integrated into the pBI121 vector (Figure 5B–D).

### 2.4. Influence of PTA Expression Cassettes with AGOs Gene Specificity on SMV Resistance

**Figure 6:**
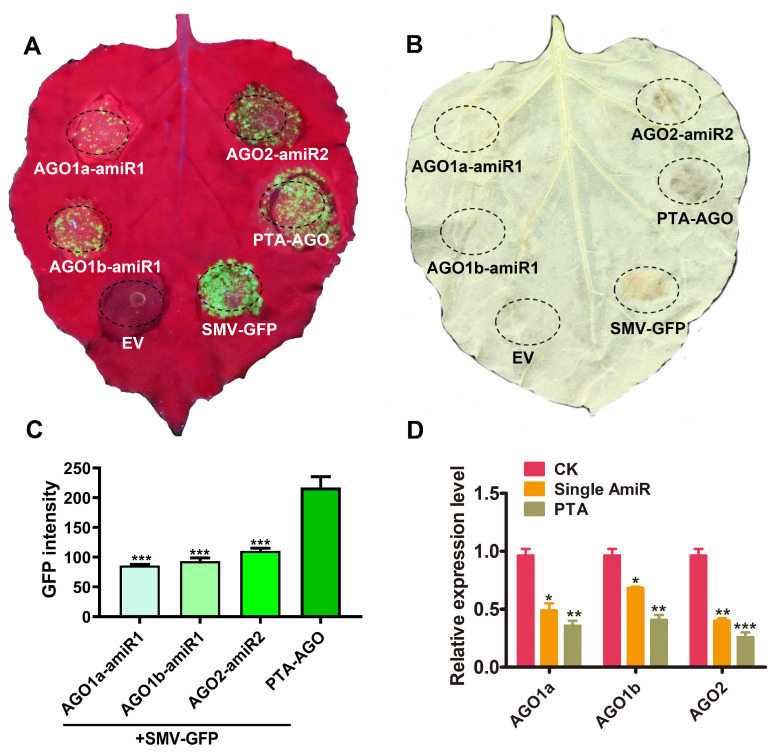
Phenotypic analysis and target gene expression in *N. benthamiana* following co-infection with either pBI121-PTA-AGO vector or individual amiRNA combined with SMV-GFP. (**A**) SMV viral accumulation; (**B**) Leaf necrosis phenotype detected by DAB staining; (**C**) GFP protein accumulation; (**D**) Relative expression level of the target gene. Significant differences between treatment and control groups were determined by Student’s *t*-test. Bars represent the standard deviation of three independent biological replicates, each with three repeats. *: *p* < 0.05, **: *p* < 0.01, ***: *p* < 0.001.

To assess whether the PTA expression cassette enhances the efficiency of RNAi, this study compared the recombinant vector pBI121-PTA-AGO, which contains three *AGO* genes, with previously selected single amiRNAs exhibiting optimal interference efficiency, namely pBI121-NbAGO1a-amiRNA1, pBI121-NbAGO1b-amiRNA1, and pBI121-NbAGO2-amiRNA2. These four recombinant vectors were individually co-infiltrated with SMV-GFP into *N. benthamiana* leaves. Phenotypic observations at six days post-infiltration revealed that leaves treated with the PTA expression cassette revealed significantly increased viral accumulation and enhanced GFP fluorescence intensity (Figure 6A). Analysis using Gel-Pro (Version 4.0.00.001) Analyzer software indicated that the fluorescence intensity of pBI121-PTA-AGO increased by 60.5%, 57%, and 49.1%, respectively, compared to the results from the single amiRNA vectors (Figure 6C). Furthermore, DAB staining revealed noticeable leaf necrosis in pBI121-PTA-AGO-treated leaves (Figure 6B), a phenomenon consistent with the changes in GFP fluorescence intensity. Target gene expression analysis demonstrated that transient expression of pBI121-PTA-AGO downregulated the expression levels of *NbAGO1a*, *NbAGO1b*, and *NbAGO2* by 25.6%, 26.7%, and 18.4%, respectively (Figure 6D). Integrative analysis of GFP fluorescence intensity, DAB staining results, SMV-GFP accumulation, and target gene expression clearly indicated that the RNAi efficiency of the PTA expression cassette is superior to that of the single amiRNAs. The PTA expression cassette effectively diminishes the expression of target genes, significantly disrupting the RNAi pathway and thereby enhancing viral infectivity in plants. These findings further confirm the involvement of the aforementioned genes in the RNAi pathway. Notably, a single amiRNA targeting *NbAGO2* alone was sufficient to induce leaf necrosis, underscoring its critical role in the RNAi mechanism and identifying it as a key AGO protein in defense against SMV.

## 3. Discussion

This study systematically investigated the functions of specific AGO family members in the *N. benthamiana*-SMV interaction system. Although the role of AGO proteins as core components of the RISC in plant antiviral immunity is well-established [12,13], the functional analysis of individual AGO members in this specific pathogen-host system remains lacking. Following inoculation with SMV-GFP, *N. benthamiana* exhibited typical viral symptoms at 14 days, including mosaic, leaf curling, and malformation, with GFP fluorescence detectable in systemic leaves. RT-qPCR results confirmed that SMV accumulated progressively in systemic leaves from day 8, reaching a high viral load by day 14 (Figure 1A,B). This infection pattern is consistent with previous reports of SMV in other solanaceous plants [14]. Notably, during the early infection stage (2 days), the expression of multiple *NbAGO* genes (*NbAGO1a*, *NbAGO1b*, *NbAGO2*, *NbAGO5*, *NbAGO7*, and *NbAGO10*) was significantly downregulated (Figure 1C), suggesting that SMV may facilitate early infection by suppressing the expression of core RNAi pathway components. This phenomenon resembles the AGO suppression strategies observed in other viruses like CMV and TMV [15,16], supporting the view that early inhibition of host RNAi core factors might be a conserved counter-defense strategy for potyviruses. As the infection progressed (6–14 days), the suppressed *NbAGO* expression gradually recovered. Specifically, the expression levels of *NbAGO1a*, *NbAGO1b*, and *NbAGO2* at 14 days were significantly higher than those in mock-inoculated controls, and their dynamic patterns closely mirrored the SMV accumulation trend (Figure 1B,C). This “suppression-recovery-upregulation” expression mode reveals a dynamic interplay between the host RNAi pathway and the virus, indicating that these three genes may play crucial defensive roles during the later stages of infection.

To clarify the evolutionary relationships and functional conservation of NbAGOs, a phylogenetic tree of AGO proteins from *N. benthamiana*, *Arabidopsis*, and *Oryza sativa* was constructed using MEGA-X. The results showed that the AGO families from these three species could be classified into three evolutionarily conserved clades. NbAGO1a and NbAGO1b clustered with *Arabidopsis* AtAGO1 and AtAGO2, whereas NbAGO2 clustered with Arabidopsis AtAGO4 and rice OsAGO17 (Figure 1D). This finding aligns with the known characteristics of AGO family evolution. Previous studies have categorized plant AGO proteins based on sequence homology into subgroups such as AGO1/5/10, AGO2/3/7, and AGO4/6/8/9, with members of different clades undertaking distinct functions within the RNAi pathway. For instance, *Arabidopsis* AtAGO1 is primarily involved in miRNA-mediated mRNA cleavage and translational repression, AtAGO2 plays a central role in antiviral defense, and AtAGO4 participates in DNA methylation and transcriptional gene silencing [17,18]. The clustering of NbAGO1a/1b with AtAGO1 suggests they may share similar miRNA-related regulatory functions, while the clustering of NbAGO2 with AtAGO4 implies its potential involvement in transcriptional-level gene silencing or antiviral defense.

Artificial microRNAs (amiRNAs) have been widely used in plant antiviral genetic engineering research due to their advantages of high safety, strong specificity, significant efficiency, and precise targeting [19]. Since the first successful use of amiRNAs to downregulate the expression of the reporter gene GFP in 2004 [20], numerous studies have confirmed that they can effectively silence specific genes in higher plants, lower plants, and unicellular organisms [21,22,23,24,25,26,27,28,29,30,31]. Addressing the application bottleneck of amiRNA technology for multi-gene simultaneous silencing-currently reported only in *Arabidopsis* [26] and *Mythimna separata* [10]. This study developed a multiplex target PTA silencing system applicable to *N. benthamiana*. Experimental results demonstrated that the PTA system reduced the transcript levels of *NbAGO1a/1b/2* by 18–27% (Figure 6D), enhanced viral GFP fluorescence intensity by 49–60% compared to the single optimal amiRNA combination (Figure 6C), and was accompanied by conspicuous DAB-positive necrotic spots. These findings directly confirm the positive regulatory roles of these three genes in defense against SMV. The enhanced silencing efficiency of the PTA system may stem from the endogenous cascade amplification effect triggered by simultaneous multi-gene silencing [32].

It is particularly noteworthy that silencing *NbAGO2* resulted in the highest viral accumulation and induced significant leaf necrosis (Figure 6B), consistent with reports concerning this gene in other virus-tobacco systems [33], indicating a central role for NbAGO2 in *N. benthamiana*’s antiviral defense. Although silencing *NbAGO1a* and *NbAGO1b* did not cause obvious necrosis, it significantly increased SMV accumulation. This aligns with the known antiviral function of AtAGO1 in *Arabidopsis*-AtAGO1 binds viral siRNAs and targets viral RNA for cleavage to inhibit viral replication [34,35].

The clustering of NbAGO1a/1b with AtAGO1 and their similar antiviral functions further support the view of functional conservation of *AGO* genes in plant antiviral defense. Notably, *N. benthamiana*’s response to some viruses (e.g., TBSV, TMV, PVX) appears to rely primarily on NbAGO2 [36], which differs from the pattern in Arabidopsis where AGO1 predominantly drives the antiviral response [34]. This species-specific response likely originates from functional diversification among AGO proteins; for example, *Arabidopsis* AGO1 is primarily involved in the endogenous miRNA pathway, whereas *N. benthamiana* NbAGO2 responds more rapidly to viral infection [37,38]. Furthermore, the viral accumulation in the group with only NbAGO2 silenced was lower than in the PTA cassette treatment group (simultaneously silencing all three genes), suggesting that NbAGO1a/1b might assist NbAGO2 in exerting its antiviral defense role, potentially by stabilizing vsiRNA complexes or enhancing the RNAi signaling system. However, the specific cooperative mechanism requires further investigation.

The PTA cassette system developed here provides a new technical approach for plant gene functional analysis and antiviral breeding. Compared to conventional single amiRNA strategies, the multiplex targeting synchronous silencing approach more effectively addresses functional redundancy issues, making it particularly suitable for analyzing members of multi-gene families. From an application perspective, the PTA cassette can be used to analyze the cooperative regulation of multiple genes within the RNAi pathway and offers new ideas for developing novel antiviral strategies. Previous antiviral research against SMV has predominantly focused on silencing single viral genes (e.g., SMV *CP* or *HC-Pro* genes) [39,40,41], but viruses can easily develop resistance through mutation.

This study validated gene function by targeting key host defense genes (*NbAGOs*), resulting in increased viral accumulation. Conversely, employing the PTA system to target multiple conserved viral genes (e.g., *VPg*, *CI*, *NIb*) could significantly reduce the probability of viral resistance emergence, thereby achieving more durable antiviral effects. Therefore, the PTA technology platform established in this study holds broad application prospects and can be further extended to antiviral research against other viruses. However, this technology has certain limitations. First, the PTA vector construction process is relatively complex, requiring multiple rounds of cloning and verification steps, which may somewhat limit its widespread application. Second, multiplex target silencing carries a potential risk of off-target effects. Although amiRNAs themselves are highly specific, the simultaneous release of multiple amiRNAs could increase the possibility of unintended silencing. Future studies could employ next-generation sequencing to comprehensively evaluate the specificity of the PTA system. Additionally, the silencing efficiency of PTA might be influenced by intracellular RNase activity and tRNA processing efficiency, and its effectiveness in different plant species may require further optimization.

In summary, this study systematically demonstrated the critical roles of NbAGO1a, NbAGO1b, and NbAGO2 in the immune response of *N. benthamiana* against SMV and successfully developed an efficient PTA multiplex gene silencing system. Experimental results proved that simultaneously silencing these three *AGO* genes significantly enhanced SMV infectivity, manifested as increased viral accumulation and aggravated symptoms, fully demonstrating the indispensable core function of AGO proteins in the plant antiviral RNAi pathway. Concurrently, this study established a complete technical workflow from key target gene screening and amiRNA sequence optimization to multiplex target vector construction, providing an important reference paradigm for subsequent plant gene function analysis and antiviral technology development. These findings enrich the theoretical system of RNAi pathway-mediated plant antiviral mechanisms and lay a crucial experimental foundation and technology platform for developing novel RNAi-based antiviral strategies. Future efforts to optimize the silencing efficiency and specificity of the PTA system and its application in the natural host of SMV, soybean, could provide innovative solutions for the green control of SMV and contribute to breeding superior SMV-resistant soybean cultivars, holding significant importance for the sustainable management of viral diseases in agricultural production.

## 4. Materials and Methods

### 4.1. Cultivation and Agents

Seeds of *N. benthamiana* were planted in a medium consisting of sterile, high-nutrient soil mixed with vermiculite in a 1:1 ratio. The plants were cultivated in the laboratory greenhouse at a temperature ranging from 23 to 26 °C and a relative humidity of 70%. The photoperiod was set at 16:8 (light: dark). For the subsequent infection experiments, seedlings at the three-leaf stage were selected. *Escherichia coli* DH5α and *Agrobacterium* GV3101 competent cells were obtained from TransGen Biotech (Beijing, China, TG-AQ601) Agents, including 2× ES-taq enzyme, Trizol agent, cDNA reverse transcription kit, and SYBR Green Master mix, *Xba*Ⅰ enzyme, T_7_ DNA ligase used for the Golden-Gate cloning were provided by Takara Bio. Primer synthesis and sequencing were conducted by Nanjing GenScript Biotech Corporation (GenScript Biotech Corporation, Nanjing, China). The expression binary vector pBI121 used in this study was maintained in the laboratory, while the T-miR159b and pGTR vectors were generously supplied by Professor Kabin Xie from Huazhong Agricultural University (Wuhan, China).

### 4.2. Construction of the Target Gene Recombinant Vector Based on amiRNA

The sequences of three RNAi core genes associated with antiviral defense (*NbAGO1a*, *NbAGO1b*, and *NbAGO2*) were obtained from the *N. benthamiana* genome database, BenthGenome Database. https://www.benthgenome.com. (Accessed: 1 May 2024). AmiRNA sequences were designed using this database, employing *Arabidopsis* miR159b as backbones. A total of three amiRNA sequences were designed for each gene, resulting in a total of nine sequences. The primers were designed to include protective sequences and restriction sites for *Xba*I and *Kpn*I, along with the amiRNA sequences and homologous miR159b sequences. Target fragments were generated through amplification and subsequently inserted into the pBI121 vector following double enzyme digestion with *Xba*I and *Kpn*I, leading to the creation of the recombinant vector pBI121-amiRNA. The accuracy of the cloning process was verified using colony PCR, enzyme digestion, and sequencing.

### 4.3. Assembly and Transient Assay of PTA Expression Cassettes in N. benthamiana

In this experiment, each amiRNA fragment was amplified using the T-miR159b plasmid as the template, and each tRNA fragment was amplified using the pGTR plasmid containing the gRNA-tRNA fusion fragment. The primers required for the construction of the PTA expression cassette are listed in Supplementary Table S1. Three single tRNA fragments and three amiR fragments were successfully amplified using the aforementioned primers and templates. PCR products were separated using 1.2% agarose gels, and the fragments of the expected size were gel-extracted. The six gel-purified fragments were mixed at a 1:1 molar ratio for Golden Gate assembly. The specific reaction system is as follows: 6 fragments obtained by gel-extraction (40–50 ng each); BSA (2 µL); 2× T_7_ DNA ligase Buffer (10 µL); *Bsa*Ι (0.5 µL); T_7_ DNA ligase (0.5 µL); and ddH_2_O (no more than 20 µL). Golden Gate assembly was performed under the following thermocycling conditions: 37 °C for 5 min (*Bsa*Ι enzyme cutting), 20 °C for 10 min (T_7_ enzyme ligation), and 20 °C for 1 h (elongation). After assembly, 180 µL of ddH_2_O was added to resolve the Golden Gate-linked products. The full-length PTA expression polycistron was amplified with the primers PTA1 and PTA13 (target length approximately 845 bp). The PTA expression cassettes were subsequently ligated into the pMD19-T vector and verified by restriction digestion, PCR, and sequencing. The verified plasmids were subcloned for the construction of the recombinant vector pBI121-PTA-AGO by ligating the PTA fragment into the pBI121 vector. All constructed vectors were confirmed by PCR, restriction enzyme digestion, and sequencing.

The recombinant plasmid pBI121-PTA-AGO was transformed into *Agrobacterium* strain GV3101. The resulting bacterial culture was used to infiltrate leaves of *N. benthamiana* at the three to four leaf stage, along with a SMV infectious clone expressing GFP. The infiltration buffer contained 1 M MES, a 1:100 dilution of MgCl_2_ solution, and a 1:1000 dilution of 200 mM acetosyringone. In parallel, control experiments were performed using individual amiRNA constructs targeting either the *NbAGO1* or *NbAGO2* gene. Additionally, empty vector controls and SMV-GFP-only treatments were included as controls.

### 4.4. RT-qPCR Analysis to Measure the Expression Level of Target Genes

In the *N. benthamiana* transient expression system, the silencing efficiency of the pBI121-PTA-AGO and pBI121-amiRNA constructs on target genes was evaluated by RT-qPCR. Leaf samples were collected at six days post-infiltration, with tissue from the infiltration zones of 10 individual leaves pooled for analysis. Total RNA was extracted using the Trizol method and reverse-transcribed into cDNA. RT-qPCR was performed on an Applied Biosystems ViiA 7 Real-Time PCR System using the PerfectStart Green qPCR SuperMix (TransGen Biotech, Beijing, China) according to the manufacturer’s protocol. Gene expression levels were normalized to *NbActin* (accession no. NM_102866.3) and relative expression was calculated using the 2^−ΔΔCt^ method. Data are presented as mean ± standard deviation from three independent biological replicates. Statistical analysis was conducted using one-way ANOVA followed by Dunnett’s post hoc test in GraphPad Prism 8 (La Jolla, CA, USA), with a p-value of less than 0.05 considered statistically significant.

### 4.5. Quantification of GFP Fluorescence Brightness

For the quantitative analysis of fluorescence in Nicotiana benthamiana leaves, Gel-Pro Analyzer software (Version 4.0, Media Cybernetics, Rockville, MD, USA). was used to convert fluorescence intensity into grayscale values for quantification. Dunnett’s multiple comparison method was employed to analyze the intergroup differences in GFP fluorescence intensity between leaves treated with individual amiRNAs and those treated with the PTA expression cassette. Ten technical replicates were set for the fluorescence intensity data of each gene. The infectious clone of SMV-GFP used in this study was constructed and validated as previously described [42]. This clone exhibits systemic infectivity and symptom presentation in *N. benthamiana* comparable to those of the wild-type virus (SMV-WT).

## 5. Conclusions

This study developed an efficient polycistronic tRNA-amiRNA (PTA) system for multiplex gene silencing, enabling functional characterization of *NbAGO1a*, *NbAGO1b*, and *NbAGO2* in antiviral defense against *Soybean mosaic virus* in *N. benthamiana*. Simultaneous silencing of these three genes significantly enhanced viral susceptibility, with individual suppression of *NbAGO2* alone sufficient to induce characteristic necrotic symptoms. The established PTA platform provides a robust tool not only for functional genomics studies in plants but also for targeted screening of RNAi components in crop improvement programs aimed at enhancing virus resistance.

## Data Availability

The data used in this research are publicly available. Each cloned gene sequences can be found at BenthGenome Database. https://www.benthgenome.com. (Accessed: 1 May 2024). The data (results) presented in this research are available in the Supplementary Materials.

## Supplementary Materials

The following supporting information can be downloaded at: https://www.mdpi.com/article/10.3390/plants14243724/s1, Figure S1. The three amiRNA sequences for the *NbAGO1a*, *NbAGO1b*, and *NbAGO2* genes; Figure S2. Sequencing results of the PTA expression cassette after Golden Gate ligation; Table S1: List of All Primer Sequences.

## References

[1] Li F., Li X., Zhao S., Pan F., Li Z., Hao Y., He J., Wang A., Kormelink R., Zhou X. (2025). Antiviral RNA interference in plants: Increasing complexity and integration with other biological processes. Plant Commun..

[2] Ali A., Shahbaz M., Ölmez F., Fatima N., Umar U.U.D., Ali M.A., Akram M., Seelan J.S.S., Baloch F.S. (2024). RNA interference: A promising biotechnological approach to combat plant pathogens, mechanism and future prospects. World J. Microbiol. Biotechnol..

[3] Betti F., Ladera-Carmona M.J., Weits D.A., Ferri G., Iacopino S., Novi G., Svezia B., Kunkowska A.B., Santaniello A., Piaggesi A. (2021). Exogenous miRNAs induce post-transcriptional gene silencing in plants. Nat. Plants.

[4] Morel J.B., Godon C., Mourrain P., Beclin C., Boutet S., Feuerbach F., Proux F., Vaucheret H. (2002). Fertile hypomorphic ARGONAUTE (ago1) mutants impaired in post-transcriptional gene silencing and virus resistance. Plant Cell.

[5] Fang X., Qi Y. (2016). RNAi in Plants: An Argonaute-Centered View. Plant Cell.

[6] Wu L., Zhang Q., Zhou H., Ni F., Wu X., Qi Y. (2009). Rice MicroRNA Effector Complexes and Targets. Plant Cell.

[7] Zhao Z., Yang S.J., Yin X.X., Yan X.L., Hassan B., Fan J., Yan L., Wang W.M. (2023). ARGONAUTE 1: A node coordinating plant disease resistance with growth and development. Phytopathol. Res..

[8] Sablok G., Pérez-Quintero A.L., Hassan M., Tatarinova T.V., López C. (2011). Artificial microRNAs (amiRNAs) engineering—On how microRNA-based silencing methods have affected current plant silencing research. Biochem. Biophys. Res. Commun..

[9] Kotowska-Zimmer A., Pewinska M., Olejniczak M. (2021). Artificial miRNAs as therapeutic tools: Challenges and opportunities. Wiley Interdiscip. Rev. RNA.

[10] Bao W., Li A., Zhang Y., Diao P., Zhao Q., Yan T., Zhou Z., Duan H., Li X., Wuriyanghan H. (2021). Improvement of host-induced gene silencing efficiency via polycistronic-tRNA-amiR expression for multiple target genes and characterization of RNAi mechanism in *Mythimna separata*. Plant Biotechnol. J..

[11] Xie K., Minkenberg B., Yang Y. (2015). Boosting CRISPR/Cas9 multiplex editing capability with the endogenous tRNA-processing system. Proc. Natl. Acad. Sci. USA.

[12] Liang C., Wang X., He H., Xu C., Cui J. (2023). Beyond Loading: Functions of Plant ARGONAUTE Proteins. Int. J. Mol. Sci..

[13] Silva-Martins G., Bolaji A., Moffett P. (2020). What does it take to be antiviral? An Argonaute-centered perspective on plant antiviral defense. J. Exp. Bot..

[14] Yang X., Li Y., Wang A. (2021). Research Advances in Potyviruses: From the Laboratory Bench to the Field. Annu. Rev. Phytopathol..

[15] Zhang X., Yuan Y.R., Pei Y., Lin S.S., Tuschl T., Patel D.J., Chua N.H. (2006). Cucumber mosaic virus-encoded 2b suppressor inhibits *Arabidopsis* Argonaute1 cleavage activity to counter plant defense. Genes. Dev..

[16] He H., Xia L., Lin Z., Li H., Ali M., Luo S., Hu Q., Zhang Y. (2025). Cell-Specific Activation and Inhibition of Conserved PTI Genes by TMV in Susceptible Tobacco. Plant Cell Environ..

[17] Wang J., Mei J., Ren G. (2019). Plant microRNAs: Biogenesis, Homeostasis, and Degradation. Front. Plant Sci..

[18] Zhang B., Zhou X., Ren Y. (2025). Host-virus molecular arms race: RNAi-mediated antiviral defense and viral suppressor of RNAi. Cell Insight.

[19] Niu Q.W., Lin S.S., Reyes J.L., Chen K.C., Wu H.W., Yeh S.D., Chua N.H. (2006). Expression of artificial microRNAs in transgenic *Arabidopsis thaliana* confers virus resistance. Nat. Biotechnol..

[20] Schwab R., Ossowski S., Riester M., Warthmann N., Weigel D. (2006). Highly specific gene silencing by artificial microRNAs in *Arabidopsis*. Plant Cell.

[21] Rabuma T., Sanan-Mishra N. (2025). Artificial miRNAs and target-mimics as potential tools for crop improvement. Physiol. Mol. Biol. Plants.

[22] Al-Roshdi M.R., Ammara U., Khan J., Al-Sadi A.M., Shahid M.S. (2023). Artificial microRNA-mediated resistance against Oman strain of tomato yellow leaf curl virus. Front. Plant Sci..

[23] Teotia S., Wang X., Zhou N., Wangm M., Liu H., Qin J., Han D., Li C., Li C.E., Pan S. (2023). A high-efficiency gene silencing in plants using two-hit asymmetrical artificial MicroRNAs. Plant Biotechnol. J..

[24] Ma Z., Wang J., Li C. (2024). Research Progress on miRNAs and Artificial miRNAs in Insect and Disease Resistance and Breeding in Plants. Genes.

[25] Bally J., Fishilevich E., Doran R.L., Lee K., Campos S.B., German M.A., Narva K.E., Waterhouse P.M. (2020). Plin-amiR, a pre-microRNA-based technology for controlling herbivorous insect pests. Plant Biotechnol. J..

[26] Zhang N., Zhang D., Chen S.L., Gong B.Q., Guo Y., Xu L., Zhang X.N., Li J.F. (2018). Engineering Artificial MicroRNAs for Multiplex Gene Silencing and Simplified Transgenic Screen. Plant Physiol..

[27] Haney C.H., Long S.R. (2010). Plant flotillins are required for infection by nitrogen-fixing bacteria. Proc. Natl. Acad. Sci. USA.

[28] Fahim M., Millar A.A., Wood C.C., Larkin P.J. (2012). Resistance to Wheat streak mosaic virus generated by expression of an artificial polycistronic microRNA in wheat. Plant Biotechnol. J..

[29] Brosseau C., Moffett P. (2015). Functional and Genetic Analysis Identify a Role for *Arabidopsis* ARGONAUTE5 in Antiviral RNA Silencing. Plant Cell.

[30] Jelly N.S., Schellenbaum P., Walter B., Maillot P. (2012). Transient expression of artificial microRNAs targeting Grapevine fanleaf virus and evidence for RNA silencing in grapevine somatic embryos. Transgenic Res..

[31] Pieczynski M., Marczewski W., Hennig J., Dolata J., Bielewicz D., Piontek P., Wyrzykowska A., Krusiewicz D., Strzelczyk-Zyta D., Konopka-Postupolska D. (2013). Down-regulation of *CBP80* gene expression as a strategy to engineer a drought-tolerant potato. Plant Biotechnol. J..

[32] Maunoury N., Vaucheret H. (2011). AGO1 and AGO2 act redundantly in miR408-mediated Plantacyanin regulation. PLoS ONE.

[33] Diao P., Zhang Q., Sun H., Ma W., Cao A., Yu R., Wang J., Niu Y., Wuriyanghan H. (2019). miR403a and SA Are Involved in *NbAGO2* Mediated Antiviral Defenses Against TMV Infection in *Nicotiana benthamiana*. Genes.

[34] Martín-Merchán A., Lavatelli A., Engler C., González-Miguel V.M., Moro B., Rosano G.L., Bologna N.G. (2024). *Arabidopsis* AGO1 N-terminal extension acts as an essential hub for PRMT5 interaction and post-translational modifications. Nucleic Acids Res..

[35] Baumberger N., Baulcombe D.C. (2005). *Arabidopsis* ARGONAUTE1 is an RNA Slicer that selectively recruits microRNAs and short interfering RNAs. Proc. Natl. Acad. Sci. USA.

[36] Odokonyero D., Mendoza M.R., Alvarado V.Y., Zhang J., Wang X., Scholthof H.B. (2015). Transgenic down-regulation of ARGONAUTE2 expression in Nicotiana benthamiana interferes with several layers of antiviral defenses. Virology.

[37] Carbonell A., Fahlgrenm N., Garcia-Ruizm H., Gilbert K.B., Montgomery T.A., Nguyen T., Cuperus J.T., Carrington J.C. (2012). Functional analysis of three Arabidopsis ARGONAUTES using slicer-defective mutants. Plant Cell.

[38] Liu P., Zhang J., Liu S., Li Y., Qi C., Mo Q., Jiang Y., Hu H., Zhang T., Zhong K. (2025). The plant signal peptide CLE7 induces plant defense response against viral infection in *Nicotiana benthamiana*. Dev. Cell.

[39] Gao L., Ding X., Li K., Liao W., Zhong Y., Ren R., Liu Z., Adhimoolam K., Zhi H. (2015). Characterization of *Soybean mosaic virus* resistance derived from inverted repeat-SMV-HC-Pro genes in multiple soybean cultivars. Theor. Appl. Genet..

[40] Kim H.J., Kim M.-J., Pak J.H., Im H.H., Lee D.H., Kim K.-H., Lee J.-H., Kim D.-H., Choi H.K., Jung H.W. (2016). RNAi-mediated Soybean mosaic virus (SMV) resistance of a Korean Soybean cultivar. Plant Biotechnol. Rep..

[41] Luan H., Liao W., Song Y., Niu H., Hu T., Zhi H. (2020). Transgenic plant generated by RNAi-mediated knocking down of soybean *Vma12* and *soybean mosaic virus* resistance evaluation. AMB Express.

[42] Bao W., Yan T., Deng X., Wuriyanghan H. (2020). Synthesis of Full-Length cDNA Infectious Clones of *Soybean Mosaic Virus* and Functional Identification of a Key Amino Acid in the Silencing Suppressor Hc-Pro. Viruses.

