# Supplementary material for: A Polycistronic tRNA-amiRNA System Reveals the Antiviral Roles of NbAGO1a/1b/2 Against Soybean mosaic virus Infection
Source: Plants (Basel). 2025 Dec 6;14(24):3724. doi: 10.3390/plants14243724 (PMC12737065; doi:10.3390/plants14243724)
Supplement: Supplementary file 1 [file plants-14-03724-s001.zip › plants-3961055-supplementary.pdf]

Supplementary Materials

Figure S1. Sequence for AGO1a, AGO1b and AGO2

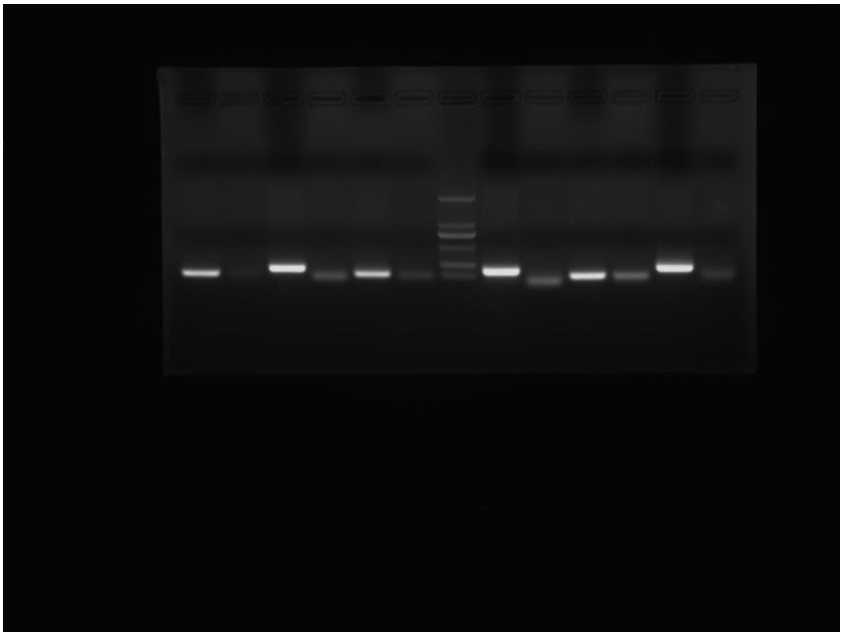

a

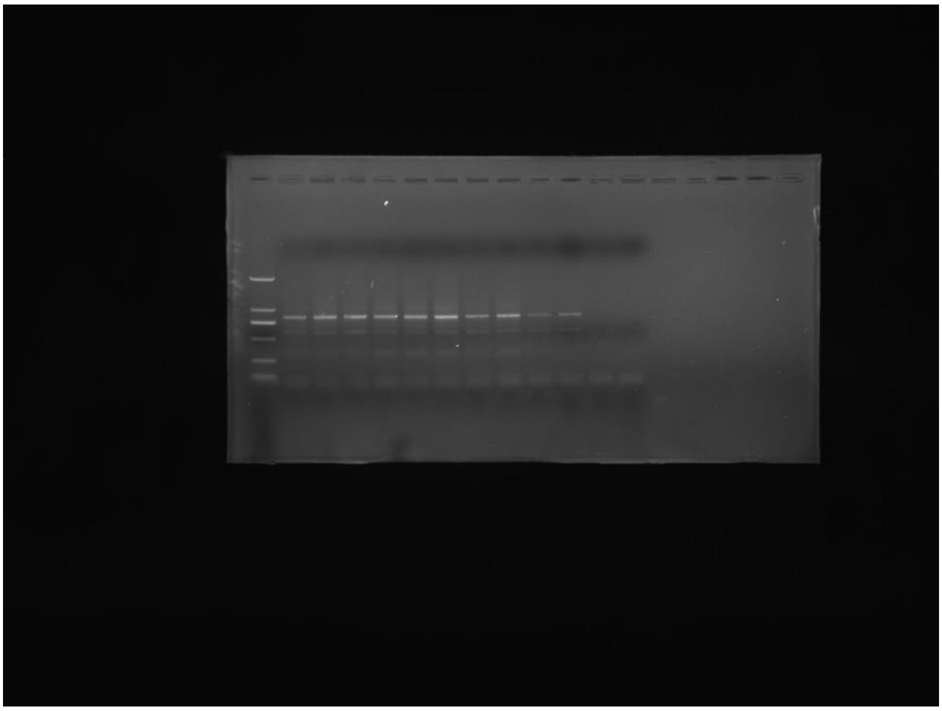

b

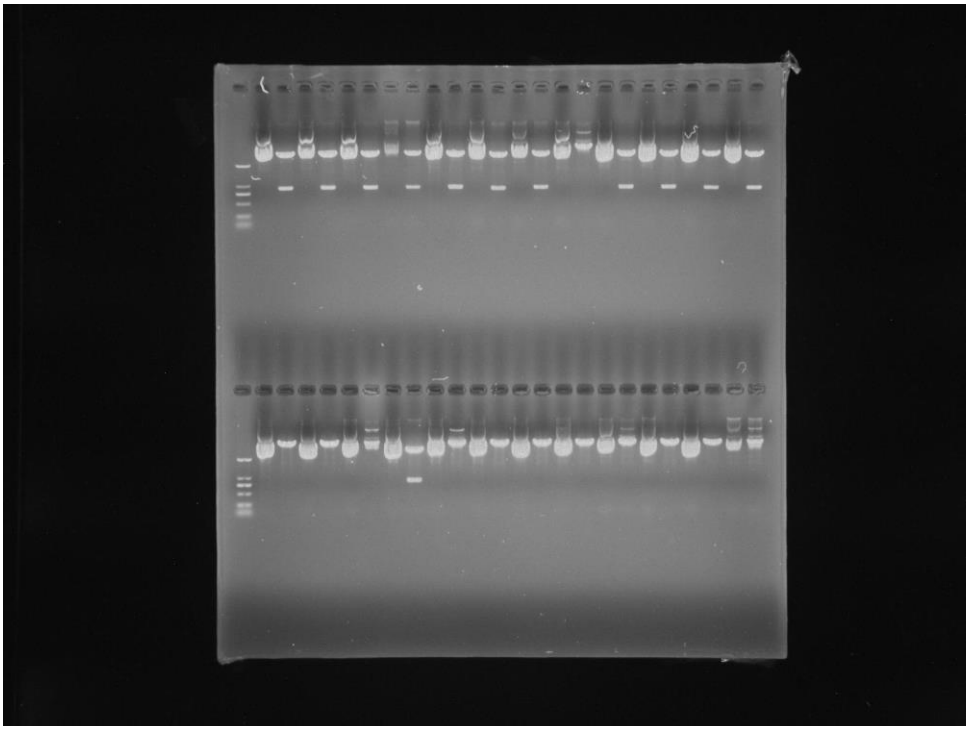

c

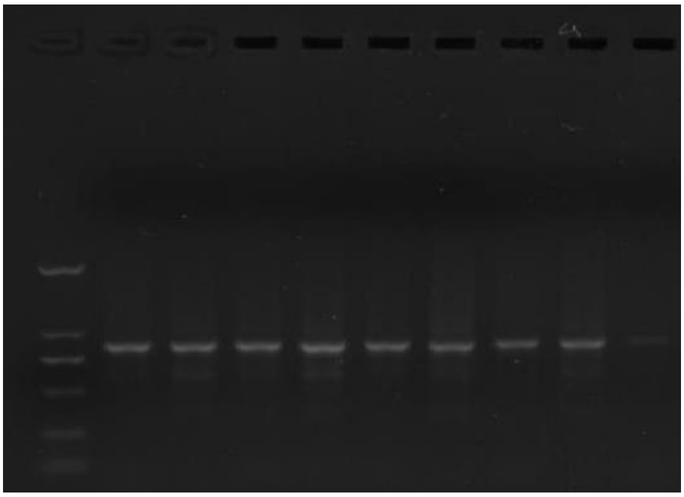

d

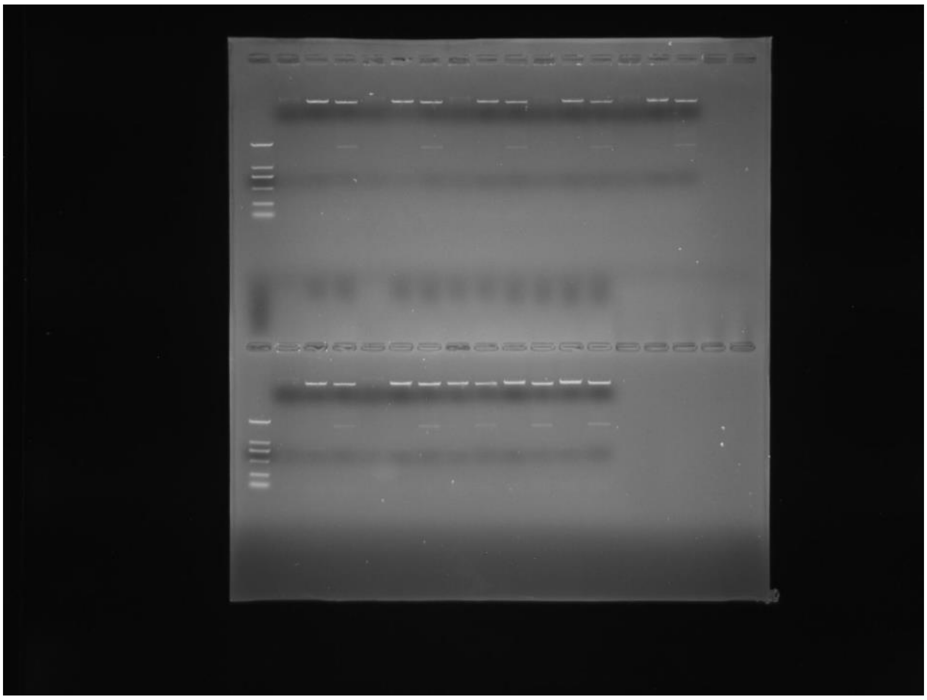

e

AGO1a-amiR1: TGCTGAGTCACTTGCTGTGTC

AGO1a-amiR1*: TCGACTCACGGAACGACACAG

AGO1a-amiR2: TCTCTCTTGAGGACGTTGGCA

AGO1a-amiR2*: TGAGAGAAAGCCTGCAACCGT

AGO1a-amiR3: TGCTGGTGGAACAATGGAGAC

AGO1a-amiR3*: TCGACCACAGTGTTACCTCTG

AGO1b-amiR1: TGCAGTGGAAGACATATCGAT

AGO1b-amiR1*: TCGTCACCGGCTGTATAGCTA

AGO1b-amiR2:TCGGTTCAGAAGCTGTGTCAC

AGO1b-amiR2*: TGCCAAGTAGTCGACACAGTG

AGO1b-amiR3: TGGCAAGTAATTAGGTCTCTG

AGO1b-amiR3*: TCCGTTCAGGAATCCAGAGAC

AGO2-amiR1: TACTCAGCGACTTCAGATCAA

AGO2-amiR1*: TTGAGTCGAGGAAGTCTAGTT

AGO2-amiR2: TCGACTTAGACCACCTAGCTT

AGO2-amiR2*:TGCTGAATAGGGTGGATCGAA

AGO2-amiR3: TATCTTCTCAGTCCTGTGGTC

AGO2-amiR3*:TTAGAAGACGCAGGACACCAG

Figure S2. Sequencing results of the PTA expression cassette after GG ligation

Red: tRNA Green: mir159b Military green:: amiRNA Protective base-*Xba*I: ggatctaga

Protective base-*Kpn*I: tcacggaggtacc gRNA: GTGGCACCGAGTCGGTGC

Pol III Terminator (TTTT..T): yellow background

ggatctagaGTGGCACCGAGTCGGTGCAACAAAGCACCAGTGGTCTAGTGGTAGAATAGT
ACCCTGCCACGGTACAGACCCGGGTTCGATTCCCGGCTGGTGCAATGCGTAACAAGCT
TGGACCTGGAGGGTTTAGCAGGGTGAAGTAAAGCTGCTAAGCTATGGATCCCATAAGC
CTTATCAAATTCAATATAATTGATGATAAGGTTTTTTTATGGATGCCATATCTCAGGAG
CTTTCACTTACCCCTTTAATGGCTTCACTCTTCATGCGTAACAATGTTGGACCAAACAA
AGCACCAGTGGTCTAGTGGTAGAATAGTACCCTGCCACGGTACAGACCCGGGTTCGAT
TCCCGGCTGGTGCACCGTCAGACACGCGAAGAGATGGAGGGTTTAGCAGGGTGAAGT
AAAGCTGCTAAGCTATGGATCCCATAAGCCTTATCAAATTCAATATAATTGATGATAAG
GTTTTTTTATGGATGCCATATCTCAGGAGCTTTCACTTACCCCTTTAATGGCTTCACTCT
TCCCGTCAGACACTGGAAGAGAAAACAAAGCACCAGTGGTCTAGTGGTAGAATAGTA
CCCTGCCACGGTACAGACCCGGGTTCGATTCCCGGCTGGTGCAGGCTAAGCTCAGCG
AGAAGTTGGAGGGTTTAGCAGGGTGAAGTAAAGCTGCTAAGCTATGGATCCCATAAG
CCTTATCAAATTCAATATAATTGATGATAAGGTTTTTTTATGGATGCCATATCTCAGGA
GCTTTCACTTACCCCTTTAATGGCTTCACTCTTCGGCTAAGCTCATGGAGAAGTAAAAA
AAggtacctccgtga

Table S1 List of All Primer Sequences

Primer Sequence (5' to 3')	Primer Name	Amplicon Length
ggatctagaGACACAGCAAGGCACTCAGCTGGA GGGTTTAGCAGGGTGAAGTAAAG	NbAGO1a-amiR1-F	204 bp
ggaggtaccGACACAGCAAGTGACTCAGCAGA AGAGTGAAGCCATTAAAGGG	NbAGO1a-amiR1-R	
ggatctagaTGCCAACGTCCGAAAGAGAGTGGA GGGTTTAGCAGGGTGAAGTAAAG	NbAGO1a-amiR2-F	
ggaggtaccTGCCAACGTCCTCAAGAGAGAGA AGAGTGAAGCCATTAAAGGG	NbAGO1a-amiR2-R	
ggatctagaGTCTCCATTGTGACACCAGCTGGA GGGTTTAGCAGGGTGAAGTAAAG	NbAGO1a-amiR3-F	
ggaggtaccGTCTCCATTGTTCCACCAGCAGAA GAGTGAAGCCATTAAAGGG	NbAGO1a-amiR3-R	
ggatctagaATCGATATGTCGGCCACTGCTGGA GGGTTTAGCAGGGTGAAGTAAAG	NbAGO1b-amiR1-F	
ggaggtaccATCGATATGTCTTCCACTGCAGAA GAGTGAAGCCATTAAAGGG	NbAGO1b-amiR1-R	
ggatctagaGTGACACAGCTGATGAACCGTGGA GGGTTTAGCAGGGTGAAGTAAAG	NbAGO1b-amiR2-F	

ggaggtaccGTGACACAGCTTCTGAACCGAGAAGAGTGAAGCCATTAAAGGG	NbAGO1b-amiR2-R	
ggatctagaCAGAGACCTAAGGACTTGCCTGGAGGGTTTAGCAGGGTGAAGTAAAG	NbAGO1b-amiR3-F	
ggaggtaccCAGAGACCTAATTACTTGCCAGAAGAGTGAAGCCATTAAAGGG	NbAGO1b-amiR3-R	
ggatctagaAAGCTAGGTGGGATAAGTCGTGGAGGGTTTAGCAGGGTGAAGTAAAG	NbAGO2-amiR1-F	
ggaggtaccAAGCTAGGTGGTCTAAGTCGAGAAGAGTGAAGCCATTAAAGGG	NbAGO2-amiR1-R	
ggatctagaGACCACAGGACGCAGAAGATTGGAGGGTTTAGCAGGGTGAAGTAAAG	NbAGO2-amiR2-F	
ggaggtaccGACCACAGGACTGAGAAGATAGAAGAGTGAAGCCATTAAAGGG	NbAGO2-amiR2-R	
ggatctagaCAGCAACCAATGAAGCCACCTGGAGGGTTTAGCAGGGTGAAGTAAAG	NbAGO2-amiR3-F	
ggaggtaccCAGCAACCAATTCAGCCACCAGAAGAGTGAAGCCATTAAAGGG	NbAGO2-amiR3-R	
ggatctagaGTGGCACCGAGTCGGT	PTA-1	187 bp
ATGGTCTCAGCCTTGCTGTGTCTGCACCAGCCGGGAAT	PTA-2	
TAGGTCTCCAGGCACTCAGCTGGAGGGTTTAGCAGGGTGA	PTA-3	120 bp
ATGGTCTCACACTTGCTGTGTCGAAGAGTGAAGCCATTAAA	PTA-4	
TAGGTCTCCAGTGACTCAGCAAACAAAGCACCAGTGGTC	PTA-5	187 bp
ATGGTCTCACCGACATATCGATTGCACCAGCCGGGAAT	PTA-6	
TAGGTCTCCTCGGCCACTGCTGGAGGGTTTAGCAGGGTGA	PTA-7	120 bp
TGGTCTCAAAGACATATCGATGAAGAGTGAAGCCATTAAA	PTA-8	
TAGGTCTCC TCTTCCACTGCAAACAAAGCACCAGTGGTC	PTA-9	120 bp
ATGGTCTCATCCCACCTAGCTTTGCACCAGCCGGGAAT	PTA-10	
TAGGTCTCCGGGATAAGTCGTGGAGGGTTTAGCAGGGTGA	PTA-11	210 bp

tcacggaggtaccTTTTTTTTTTTTCGACTTAGACC ACCTAGCTTGAAGAGTGAAGCCA	PTA-12	
tcacggaggtaccTTTTTT	PTA-13	835 bp
GTCAAGGCTGGGTTTGCTGG	NbActin-qPCR-F	200 bp
CCCACGTAGGCATCTTTCTG	NbActin-qPCR-R	
GTAAGATTGTAGAGGGACAGAGA	NbAGO1a-qPCR-F	194 bp
AACTTGAGCAAGCTTCTCACTG	NbAGO1a-qPCR-R	
AGAAAGTTGGCGTGGGTTCTAT	NbAGO1b-qPCR-F	178 bp
TATCTTTACACGGCCAGCATCA	NbAGO1b-qPCR-R	
ATGTGAAATGGTACGGGCTGAA	NbAGO2-qPCR-F	176 bp
CAACAAGGTTCCACTGGCATTT	NbAGO2-qPCR-R	

Note: The sequences highlighted in red represent the *Xba*I restriction site, and those in green represent the *Kpn*I restriction site.
